# Data collection systems for active safety surveillance of vaccines during pregnancy in low- and middle-income countries: developing and piloting an assessment tool (VPASS)

**DOI:** 10.1186/s12884-023-05417-8

**Published:** 2023-03-13

**Authors:** Maria Belizán, Federico Rodriguez Cairoli, Agustina Mazzoni, Erin Goucher, Sabra Zaraa, Sarah Matthews, Verónica Pingray, Andy Stergachis, Xu Xiong, Mabel Berrueta, Pierre Buekens

**Affiliations:** 1 Instituto de Efectividad Clínica y Sanitaria (IECS), Dr. Emilio Ravignani 2024 (C1014CPV), Buenos Aires, Argentina; 2 Tulane University School of Public Health and Tropical Medicine, New Orleans, LA 70112 USA; 3 School of Pharmacy, University of Washington, Seattle, WA 98195 USA; 4 School of Public Health, University of Washington, Seattle, WA 98195 USA

**Keywords:** Safety surveillance, Data collection system, DHIS2, INDEPTH, Global Network, Pharmacovigilance, Pregnancy, Vaccines, Maternal immunization, COVID-19

## Abstract

**Background:**

There is an urgent need for active safety surveillance to monitor vaccine exposure during pregnancy in low- and middle-income countries (LMICs). Existing maternal, newborn, and child health (MNCH) data collection systems could serve as platforms for post-marketing active surveillance of maternal immunization safety. To identify sites using existing systems, a thorough assessment should be conducted. Therefore, this study had the objectives to first develop an assessment tool and then to pilot this tool in sites using MNCH data collection systems through virtual informant interviews.

**Methods:**

We conducted a rapid review of the literature to identify frameworks on population health or post-marketing drug surveillance. Four frameworks that met the eligibility criteria were identified and served to develop an assessment tool capable of evaluating sites that could support active monitoring of vaccine safety during pregnancy. We conducted semi-structured interviews in six geographical sites using MNCH data collection systems (DHIS2, INDEPTH, and GNMNHR) to pilot domains included in the assessment tool.

**Results:**

We developed and piloted the “VPASS (Vaccines during Pregnancy – sites supporting Active Safety Surveillance) assessment tool” through interviews with nine stakeholders, including central-level systems key informants and site-level managers from DHIS2 and GNMNHR; DHIS2 in Kampala (Uganda) and Kigali (Rwanda); GNMNHR from Belagavi (India) and Lusaka (Zambia); and INDEPTH from Nanoro (Burkina Faso) and Manhica (Mozambique). The tool includes different domains such as the system’s purpose, the scale of implementation, data capture and confidentiality, type of data collected, the capability of integration with other platforms, data management policies and data quality monitoring. Similarities among sites were found regarding some domains, such as data confidentiality, data management policies, and data quality monitoring. Four of the six sites met some domains to be eligible as potential sites for active surveillance of vaccinations during pregnancy, such as a routine collection of MNCH individual data and the capability of electronically integrating individual MNCH outcomes with information related to vaccine exposure during pregnancy. Those sites were: Rwanda (DHIS2), Manhica (IN-DEPTH), Lusaka (GNMNHR), and Belagavi (GNMNHR).

**Conclusion:**

This study's findings should inform the successful implementation of active safety surveillance of vaccines during pregnancy by identifying and using active individual MNCH data collection systems in LMICs.

**Supplementary Information:**

The online version contains supplementary material available at 10.1186/s12884-023-05417-8.

## Introduction

There is an urgent need for active safety surveillance to monitor vaccine exposure during pregnancy in low- and middle-income countries (LMICs). Passive surveillance systems, the most commonly used method in LMICs, do not ensure an unbiased evaluation of maternal and neonatal outcomes after drug or vaccine exposures during pregnancy [1–3]. In contrast, active safety surveillance involves the systematic collection of information on the presence or absence of adverse events and other relevant information within a defined group of people, such as pregnant people [2]. The launch of new vaccines for the immunization of pregnant people requires adequate monitoring to achieve their main objective of decreasing maternal and newborn morbidity and mortality in a safe and effective manner [2, 4, 5].

Existing maternal, newborn and child health (MNCH) data collection systems could serve as platforms for post-marketing active surveillance of maternal immunization safety in LMICs. In particular, these systems could provide background rates of health outcomes of interest as well as data on individuals exposed (and/or unexposed) to the vaccine to carry out active safety surveillance of MNCH outcomes [6, 7].

Considering these needs, we conducted a scoping review to identify MNCH data collection systems that can support active safety surveillance for novel maternal vaccines. Through this review, eight potential systems were identified [8]. Out of the total eight data collection systems identified, three were prioritized by a consensus of experts in the field, based on seven different dimensions: governance, system design, system management, data management, data sources, outcome measurement, and data quality. Specifically, these three systems selected were: District Health Information Systems (DHIS2), The International Network for the Demographic Evaluation of Populations and Their Health (INDEPTH), and The Global Network Maternal Newborn Health Registry (GNMNHR) [8].

Briefly, DHIS2 is a free, open-source custom software platform that collects, analyzes, visualizes, and shares data and that has been used mainly as a health management information system. It supports aggregate and individual-level data in more than 75 LMICs, including national-level health programs. Recently, it has been used by many LMICs for the follow-up of patients diagnosed with COVID-19 and as a register of COVID-19 vaccination [9]. The INDEPTH network is currently composed of more than forty-member health research centers that collect data through Health and Demographic Surveillance Systems (HDSSs) in 19 LMICs in Africa, Asia, and Oceania [10]. GNMNHR is a prospective, population-based registry implemented in areas of low-resource countries for research purposes. Nearly 370,000 mothers and their infants have been enrolled in this registry from 2008 until June 2021 [11, 12].

Although general information on these systems has been described [8], possible differences in the operational use at different geographical sites still need to be addressed. For instance, although many sites currently use DHIS2 to track individual data, not all use the system to track individual MNCH information, which is essential to support active safety surveillance [9]. Likewise, in the case of INDEPTH, although all member centers track pregnancies, births, and deaths, some sites routinely collect more parameters and more accurate maternal and child data [13, 14].

Since some major differences exist between sites using the same system, it becomes crucial to conduct a more detailed assessment of these sites to facilitate the appropriate selection of specific centers for active safety surveillance in LMICs. As far as we know, there is not yet an assessment tool designed to evaluate potential data collection systems on-site that would support active safety surveillance of vaccines used by pregnant persons in LMICs. Nevertheless, some widely-used frameworks and guidelines in the field that could guide the development of a new tool adapted to this purpose are available [15, 16].

With the purpose of contributing to the identification of sites that can support active surveillance of vaccination during pregnancy in LMICs, this study had two objectives. First, to develop an assessment tool that can evaluate different domains of perinatal data collection systems potentially contributing to active safety surveillance of vaccines in pregnant people in LMICs. Second, to pilot this tool in sites using the MNCH data collection systems already mentioned. This study is part of a landscape analysis led by Tulane University, the University of Washington, and the Institute for Clinical Effectiveness and Health Policy (IECS).

## Methods

### Landscape analysis methodological process

**Fig. 1:**
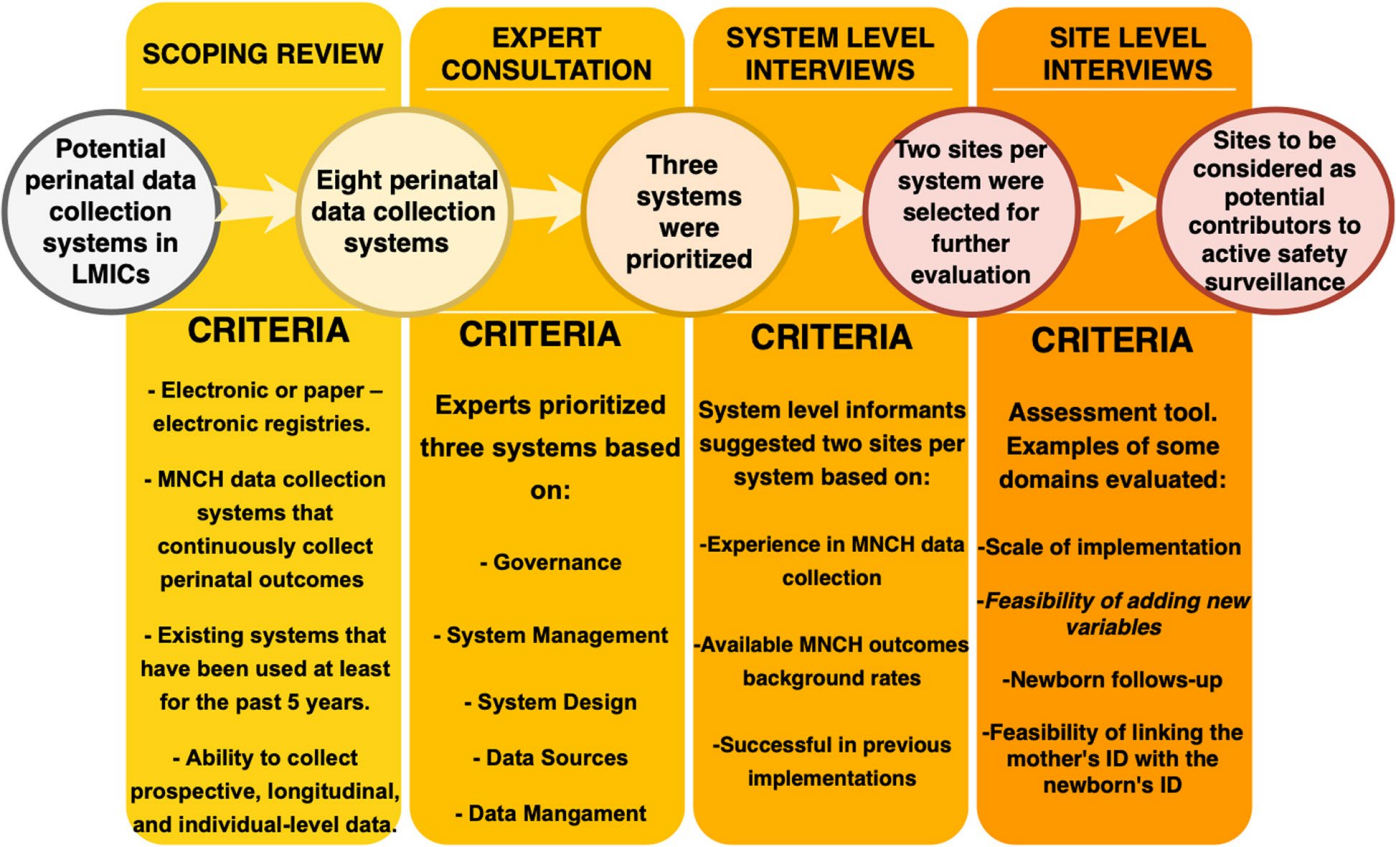
Landscape analysis’ steps and summary of findings

Figure 1 summarizes the methodology and processes performed throughout the landscape analysis to identify sites to be evaluated. During previous steps of this landscape analysis, we performed a scoping review and an expert consultation to select perinatal collection systems in LMICs. The systems selection criteria during those steps are summarized in Fig. 1, and the findings are described elsewhere [8]. Only three systems that could perform MNCH data collection at the individual level were prioritized during those steps.

This manuscript describes the finding's last steps of the landscape analysis including System Level interviews and Site Level interviews.

### Assessment tool development

We conducted a rapid review of the literature to identify frameworks on population health or post-marketing drug surveillance that could serve as the basis for developing a tool to evaluate country sites that could support active monitoring of vaccine safety during pregnancy. The inclusion criteria included any guide, publication, or review that describes tools that assess health data collection systems for population or post-marketing drug active surveillance. In addition, we prioritized those frameworks developed or published by international and known public health research organizations. Records detailing tools that focus only on one-hospital medical record system assessment or health information systems mainly used in high-income countries were not considered. We also excluded frameworks focused on specific disease surveillance assessments (for instance, HIV/AIDS, malaria, or tuberculosis). Topics related to passive safety surveillance were not considered since it is not an object of our study.

We ran a search strategy in PubMed/Medline on September 15th, 2020, that contains the following terms: (Framework[tiab] OR assessment tool[tiab]) AND (Health information systems[tiab] OR safety surveillance[tiab]). We applied the "Humans" filter and searched for studies published in the last twenty years. In order not to lose any record of interest, the research group also carried out a gray literature search through Google Scholar using the following words: "framework", "health information system", and "assessment tool".

A total of 156 records were identified through the literature search strategy. After evaluating by title and abstract, four frameworks that met the eligibility criteria were identified and prioritized: The Center for Disease Control (CDC) Updated Guidelines for Evaluating Public Health Surveillance Systems [15]; Guide to Active Vaccine Safety Surveillance: Report of The Council for International Organizations of Medical Sciences (CIOMS) Working Group on vaccine safety [16]; MEASURE Evaluation: Strengthening Health Information Systems in LMICs [17] and World Health Organization (WHO) COVID-19 vaccines: Safety surveillance manual [18]. The last two frameworks were identified through the gray literature search. After a detailed examination, the research team prioritized specific subjects described in these guides consistent with the objectives of this tool. That was because the guidelines identified also included domains outside our study's interest. For example, the CDC [15] and MEASURE Evaluation [17] guidelines assess not only vaccine surveillance domains but also other surveillance components in population health. If some subject was mentioned and described in more than one of these four sources, the research team agreed to take as reference the one that best suited the purposes of this research.

The research group then formulated different domains to group them. The sum of these domains constituted the entire assessment tool. A first draft of the assessment tool was then prepared and evaluated by a group of subject matter experts who constituted our project’s Scientific and Technical Advisory Group (STAG). After implementing their feedback, a definitive validated version was obtained and is described in detail in the results section. The tool was named “VPASS Assessment Tool” (New **V**accines during **P**regnancy—Sites Supporting **A**ctive **S**afety **S**urveillance Assessment Tool).

### Assessment of sites using MNCH data collection systems through semi-structured interviews: piloting the VPASS Assessment Tool.

We carried out a qualitative study using semi-structured interviews to pilot the VPASS Assessment Tool. Stakeholders responsible for managing and/or using the data collection systems on-site were identified and interviewed via online audio conferencing. The data collection took place from April to August 2021.

Key informants were identified using purposive sampling through the recommendations of the research team and members of the STAG. Central-level systems informants—key staff in charge of system development, management, and coordination—were selected based on their knowledge and experience managing one of the three data collection systems.

Site-level informants were experts, managers, or directors of the system at the country site. The decision to include two sites per system was taken deliberately based on the importance of piloting the tool in more than one site per system that were feasible to reach and meet the following criteria: experience collecting MNCH data, availability of MNCH outcomes background rates, and success in previous data collection, management, or analysis implementations.

The selection of the sites was agreed between the central-level informants and the research team.

Kampala (Uganda) and Kigali (Rwanda) were the sites selected for DHIS2, while Belagavi (India) and Lusaka (Zambia) were those for GNMNHR. Due to the decentralized nature of the INDEPTH, a central-level interview could not be performed. Thus, STAG members were requested to suggest sites following the same criteria as for the other systems. As per their recommendation the informants for INDEPTH were one in Nanoro (Burkina Faso), and the other in Manhica (Mozambique).

Semi-structured questionnaires were designed to obtain key information using the different domains of the assessment tool as a guide. We used the same set of open-ended questions in each of the domains. We adapted the questions according to the information we already had before conducting the interviews in an attempt to validate the information we already had or to go deeper into data we did not have. We did not follow a specific order of questions but adapted them according to each interviewee's narration.

Interviews were conducted virtually using the Zoom cloud-based video communications app [19]. Interviews were led by a researcher with experience in qualitative interviews (MB) and researchers with experience in MNCH and surveillance (FRC and AM). In addition, a research assistant took the role of note-taker (EG). Verbal consent was obtained before each interview. Interviews were conducted in English and lasted between 35–60 min.

Meetings were audio-recorded, and each interview was then transcribed verbatim in preparation for the analysis. Transcripts were coded using the software for qualitative analysis Atlas TI version 8 [20]. A thematic analysis was conducted for each relevant domain from the assessment tool. After analyzing all the data, the research team sent the results to the interviewees via email for their review.

## Results

### VPASS Assessment tool domains

The assessment tool included general system domains such as data management policies, internal data quality monitoring, and system’s purpose, and specific site-level domains such as the scale of implementation, data capture, and experiences in MNCH data collection. Table 1 describes the assessment tool domains.

**Table 1:** VPASS Assessment tool domains for evaluating data collection system-sites to support active safety surveillance for vaccine exposures during pregnancy in LMICs

**Domain**	**Brief description and Reporting suggestion**
Purpose of the system	Description of the uses of the data obtained by the system such as surveillance, research and/or, clinical care
Geographical location	Name of the site or sites currently using the system for MNCH data collection such as country, city, region or district
Scale of implementation	System coverage scale at the site such as National, Provincial, Regional, district, etc
Experience in MNCH data collection	Time of system use at the site in months or years
MNCH variables under surveillance	List of MNCH variables/outcomes collected routinely: GAIA Maternal and Neonatal Outcome Case Definitions [21]
Data capture place	Where the data collection takes place such as at health facility or at community level
Level of data disaggregation	Whether the data is aggregated or disaggregated (individual data)
Method and frequency of data collection	If the Data is collected prospectively or retrospectively. If it is collected routinely and without interruption or only under specific circumstances
Data capture format	Whether the data is captured onto an electronic form and/or paper based
Data standardization on MNCH variables	If the system and site comply with applicable standards for data formats and coding schemes; and explain if relevant
Newborn follow-up	Whether they follow up newborns and for how long (in days, weeks, or months)
Representativeness	If they follow the whole population or if there are groups that are systematically excluded from the reporting system. Explain if relevant
Reliability	Possibility to link between identifier number (ID) of the mother and identifier number (ID) of the newborn
Integration with other registers	Capability to integrate data with other health registers such as registers that provide vaccine-related information (i.e., vaccine type, number of doses, injection site)
Feasibility of adding new variables	Whether the site can implement new healthcare coding standards (e.g., ICD-10) or new MNCH variables; and explain if relevant
Data management policies	If they comply with applicable federal and state statutes and regulations
Data confidentiality	Whether the procedures to ensure patient privacy and data confidentiality are in place
Staff training	Whether those operating the system receive training and what type of training
Site experience in MNCH active safety surveillance	Explain if the site has performed MNCH active safety surveillance
Availability of baseline data	Report if the site has baseline data for potential use when conducting active safety surveillance
Internal data quality monitoring	If the site or system perform a real-time internal data validation during the data entry process; include explanation if relevant
External data quality monitoring	Whether they perform external/audits assessments for data validation; and how
Data quality and data completeness (*)	If data quality and data completeness is appropriate enough to the point, they can support active safety surveillance
Timeliness (*)	Whether the time between crucial data reporting steps appropriate is appropriate

(*) Preferably, these domains should be evaluated objectively (direct observation), and not through interviews. For this reason, they will not be addressed in this study

### Assessment of sites using MNCH data collection systems

The research team first interviewed central-level referents from DHIS2 at the University of Oslo (*n* = 2) and GNMNHR at RTI International (*n* = 1). Then, local key informants from Kampala, Uganda (*n* = 1) and Kigali, Rwanda (*n* = 1) were interviewed for DHIS2; and from Belagavi, ​​India (*n* = 1) and Lusaka, Zambia (*n* = 1) for GNMNHR. For INDEPTH, local key informants from Nanoro, Burkina Faso (*n* = 1) and Manhica, Mozambique (*n* = 1) were interviewed.

Table 2 summarizes the findings for each site. The domains "data quality and data completeness" and "timeliness" were not addressed in this study since they should be answered objectively by evaluating information produced by the systems on-site and not through interviews. Also, the main findings, challenges, and possible important strengths that have been highlighted by the informants during the interviews are described narratively.

**Table 2:** VPASS Assessment tool pilot in six sites using three MNCH data collection -summarized information from interviews

Assessment tool domain	MNCH DATA COLLECTION SYSTEM					
	**DHIS-2** **Purpose:** Clinical Care		**INDEPTH** **Purpose:** Clinical Care		**GNMNHR** **Purpose:** Research	
Geographical location	Rwanda	Uganda	Burkina Faso	Mozambique	Zambia	India
Scale of implementation	National Level	National Level	District level (Nanoro)	District level (Manhica Health Research Center)	Province level (Lusaka)	Village level (Belagavi)
MNCH variables under surveillance	See Supplementary Material					
Data capture place	Health facilities and community level	Health facilities and community level	Community level only	Health facilities and community level	Mainly at health facilities	Health facilities and community level
Level of data disaggregation	Individual data	Only aggregated data	Only aggregated data	Individual data	Individual data	Individual data
Methods and frequency of data collection	Prospectively and regularly	Prospectively and regularly	Retrospectively and regularly	Retrospectively and regularly	Prospectively but only for research purposes	Prospectively but only for research purposes
Data capture format	Mainly electronic	Electronic and paper based	Mainly paper based (Electronic only during specific projects)	Mainly paper based (Electronic only during specific projects)	Switching from paper based to electronic format (through REDCap)	Switching from paper based to electronic format (through REDCap)
Data standardization on MNCH variables	Yes. Based on ICD 10 standard code	Yes. Based on ICD 10 standard code	Yes. Based on ICD 10 standard code	Yes. Based on ICD 10 standard code	Not based on ICD or MedDRA standard codes	Not based on ICD or MedDRA standard codes
Newborn’s follow up	Routine follow-up of newborns for 6 months postpartum	Newborn’s individual information is not routinely collected after delivery	Newborn’s individual information is not routinely collected after delivery	Routine follow-up of newborns for 6 months postpartum. (Childs followed up on regular rounds every six months)	Routine follow-up of newborns for 6 weeks postpartum	Routine follow-up of newborns for 6 weeks postpartum
Data management policies	All sites comply with applicable data management national regulations					
Data confidentiality	All sites use encryption of personal data					
Data quality monitoring	All sites perform internal and external monitoring for data quality					
Representativeness	In all sites no specific pregnant population is systematically excluded					
Reliability	Possible to link Mother ID with newborn ID	It is not yet possible to link Mother ID with newborn ID	Possible to link Mother ID with newborn ID	Possible to link Mother ID with newborn ID	Possible to link Mother ID with newborn ID	Possible to link Mother ID with newborn ID
Integration with other registers	Yes. It is possible to integrate MNCH individual data with electronic immunization registries [22]	Not yet possible for MNCH data	Not yet possible for MNCH data	Yes. It is possible to integrate MNCH individual data with local medical registries at health facilities	Not routinely (it requires permission from RTI)	Not routinely (it requires permission from RTI)
Feasibility of adding new MNCH variables	Yes, it is possible	Yes, it is possible	Not enough information	Yes, it is possible	Yes, it is possible	Yes, it is possible
Staff training	Yes. All sites provide continuous training to their staff					
Experience in MNCH individual data collection	It collects MNCH individual data since 2017 (previously, only aggregate data were collected)	Not applicable since it only collects aggregated information	Not applicable since it only collects aggregated information	It collects MNCH individual data since 2016	It collects MNCH individual data since 2008, when the Maternal Newborn Health Registry (MNHR) was created	
Baseline data available	Yes, for all the variables collected	Yes, but only aggregated information for maternal and neonatal deaths	Yes, but only aggregated information for maternal and neonatal deaths	Yes, for all the variables collected	Yes, for all the variables collected	Yes, for all the variables collected
Previous experience in MNCH active safety surveillance	No previous experience	No previous experience	No previous experience	No previous experience	Yes, but only within research context	Yes, but only within research context

*Note*: These data were collected in August 2021. At the time of publication of this manuscript there may have been changes on some of the sites for some of the reported findings

### DHIS2 sites

DHIS2 is currently used at the national level in the Rwanda and Uganda sites. However, most data collected by DHIS2 Uganda is aggregate data, though there is some individual surveillance data for notifiable diseases, maternal and perinatal audits, and to some extent, tuberculosis. In particular, the only maternal and perinatal health data collected by DHIS2 in Uganda is aggregate data of maternal and perinatal deaths. In Rwanda, individual data collection was introduced at the national level in 2014 with the DHIS2 Tracker, which is an events-based system. Individual data collection for maternal and neonatal variables began in 2017: previously aggregate information was collected on the number of births and the number of newborns. The DHIS2 Tracker is also used for the national immunization registry that follows the mothers and children up until the child receives their last recommended vaccination.

Concerning the local context, the informant from Uganda highlighted the challenges related to data collection on the site. While this information is being collected on paper, it is not being entered into the platform on time. Sites may not have access to technology resources like Internet connectivity or the availability of skilled analysts.

*“I think the biggest challenge we have is related to real-time data capture. I say this because vaccination is happening in almost every district and its wide. But we are not able to capture data in real-time. So, we are to enter it into the paper and then turn it into data entry, so we are not able to scale it to all the facilities”*

*"You need to have people on the ground to be able to enter data, it requires technological support in terms Internet connectivity… So then also the skill set and skill levels, can all these health workers be trained to use the new technology” (Uganda, DHIS2).*

In the case of Rwanda, the informant commented that one of the main challenges they encountered was the resistance to the switch from paper to electronic data collection. However, Rwanda has made progress in this area. Indeed, the informant talked about the benefits of using DHIS2 during the COVID-19 pandemic for individual data collection and data analysis.

The Rwanda informant highlighted the wide acceptance of DHIS2 at the national level, both in the private and public sectors:

*"Since the DHIS2 implementation at the national level in 2012, we are using it at all levels, including private facilities. So, all public facilities are using it. Private facilities are using it and the other sites that are linked to the Ministry of Health. The ministry really encourages all partners and stakeholders to support the HMIS system, which is DHIS2" (Rwanda, DHI2)*

### GNMNHR sites

The central-level interviewee for the GNMNHR shared that the GNMNHR's activities are entirely research-based, and the registry does not provide country surveillance nor is the registry linked to medical records. At each site, one institution oversees that data collection, and its data managers and field supervisors provide quality assurance and registry administrators. The GNMNHR is representative of its catchment areas, as the field workers enumerate all households in the catchment areas for which they may conduct annual surveys. This type of data collection allows for pregnancies and deliveries to be captured even if the pregnant person does not attend a health facility. Data may also be collected from the local health centers.

The system-level researchers work to review the data and identify outliers or variables that are not being collected uniformly across the sites. Finally, the stakeholder shared that all the GNMNHR sites have recently transitioned from paper-based data collection to electronic data collection using REDCap and previously collected data is being transferred to the new electronic platform.

The Zambian stakeholder shared that data is collected from ten different health centers in the country, and every individual who attends one of these clinics during their pregnancy is enrolled into the registry. Even though institutional deliveries are increasing in Zambia, the GNMNHR engages with the community they work in to promote enrollment into their registry to ensure its representativeness of the catchment area.

*"It's not a national registry. It's within Lusaka, but within that vast Lusaka, it covers quite a number of 10 health centers, which is quite representative of the country. Most of the information collected can be extrapolated to a predictable extent." (Zambia, GNMNHR)*

The Indian stakeholder emphasized the representativeness of the GNMNH registry. Overall, enrollment into the registry in Belagavi is above 95%, and the registry does not exclude any individuals unless they do not consent. The MNH registry in Belagavi, India conducts an annual household survey to identify people who are likely to conceive that year. The auxiliary social health advocates (ASHAs) are the field workers who conduct the initial survey and the follow-ups with the identified people. When a person is confirmed to be pregnant, an ASHA will advise them to visit the closest health facility to be enrolled in the registry. This system allows for very early pregnancy identification. Fifty percent of the pregnancies in the MNH registry are registered before 8 weeks, and as much as 95% of the pregnancies are registered before 12 weeks.

*“If we just look into the process, it will start from the annual household survey. Our auxiliary social health advocates visit houses to see to it that any pregnancy should not get missed. So, we will register all the pregnant women. Additionally, we follow them up not only to delivery, but 42 days post-delivery” (Belagavi, GNMNHR)*

### INDEPTH sites

Interviews about the INDEPTH network were only obtained from site-level stakeholders. Stakeholders from Burkina Faso and Mozambique emphasized the decentralized nature of the network and the independence of their own country's systems. In both countries, data collection is conducted by field workers. This type of field data collection is necessary as the percentage of people who deliver at health institutions or attend antenatal care is low. Also, the network is not linked with health facilities' information.

In Burkina Faso, field workers are present in every village the center covers, and once a pregnancy is reported it is confirmed by a supervisor. In Mozambique, pregnancies are registered both by field workers and community informants.

*"As you may know, not all of the population or women will attend the health facility for the national health system; we may miss some of the population. So actually, HDSS, uh, let's see, a coverage gap and what we are collecting is actually births and pregnancies." (Burkina Faso, INDEPTH)*

*"So, for some group of neighborhoods, we identified a key informant. So we visit them in order to collect the information or events that happened between our rounds. They collect information about their neighbors or their households or people who live in the neighborhood,". (Mozambique, INDEPTH)*

The INDEPTH in Mozambique can collect data on mothers and neonates in several ways, including the bi-annual household visits conducted by the field workers, the village informants, and their free call center. Community key informants also aid in data collection, and staff from the center visit with the informants bi-weekly to collect additional information that may have been missed. The Mozambican stakeholder mentioned that challenges in using INDEPTH were difficulties adapting the system to meet the specific health needs, as when the Center began to introduce longitudinal data and enumerate households. Finally, data collection is mainly paper based for INDEPTH in Burkina Faso and Mozambique, with both sites citing resources as a barrier to implementing electronic data collection.

## Discussion

This manuscript describes the findings of the last step of a landscape analysis that aimed to assess LMIC sites’ readiness for integrated active safety surveillance for novel vaccines during pregnancy (see Fig. 1). During the first stages of the analyses three data collection systems to support active safety surveillance in LMICs were prioritized (DHIS2, GNMNHR and INDEPTH). In the last stage, we developed the VPASS Assessment Tool for the evaluation of essential domains of these MNCH data collection systems. Then, six sites using one of these systems were selected and assessed by piloting the VPASS Assessment Tool. We implemented the assessment tool through interviews with stakeholders at the system and the site levels.

When comparing the implementation of the tool among sites, similarities were found, such as data confidentiality, data management policies, and data quality monitoring. However, differences were found regarding the scale of implementation, data collection processes, newborns follow-up, and the integration with other systems. The capacity of routinely collect MNCH individual data was essential to define the site’s capacity to support active safety surveillance. As a result, sites located in Rwanda (DHIS2), Manhica (Mozambique, INDEPTH), Lusaka (Zambia, GNMNHR), and Belagavi (India, GNMNHR) could be considered to support active safety surveillance of vaccinations during pregnancy.

Ideally, data collection systems should be capable of electronically integrating individual MNCH data with other health records. Optimal active safety surveillance requires the electronic integration of information related to vaccine exposure during pregnancy with the collected MNCH outcomes. Recently, DHIS2 has implemented a module that allows recording participants’ data when receiving the COVID-19 vaccination in 42 countries [23]. With this module, it is possible to collect information about whether the person receiving the vaccine is pregnant and, if so, record the respective trimester of pregnancy. This module is operational in Rwanda and Uganda DHIS2 sites. Of note, the module does not collect individual MNCH information per se but need to be integrated with the individual MNCH data already collected. Currently, only the site in Rwanda is prepared to link individual MNCH collected data with the information recorded in this immunization registry [23].

Other potential sites also could support active safety surveillance. In particular, the Palestine site for DHIS2 has vast experience in electronically collecting individual MNCH data [24] Likewise, some sites of the INDEPTH system have been working to improve the MNCH data collected through population surveillance health surveys [25]. In particular, INDEPTH sites in Bangladesh, Ethiopia, Ghana, Guinea-Bissau, and Uganda have been implementing the Every Newborn Action Plan (ENAP), intending to end preventable newborn and stillbirth deaths. The work carried out to improve data quality on some MNCH variables has been recently published [26].

Some LMICs currently use more than one data collection system to record MNCH information. Certain systems perform better at collecting data at the community level (INDEPTH). In contrast, others at the health facility level (DHIS2), so differences between systems need to be considered in any effort to integrate information. Thus, unifying data from different data collection system sources could improve active safety surveillance.

The strengths of our study include developing and using of a new tool to assess data collection systems in potential sites to evaluate their ability to support vaccine active safety surveillance in pregnant persons. This unique approach allowed for collecting stakeholders’ valuable insights on their use and experience of the systems, perceived strengths, and weaknesses. Both levels of data collection ensured that we captured critical information from the development to the implementation process and sites’ experiences collecting MNCH data. After the first stages of the data processing were completed, we shared and validated our findings with the interviewees. Also, this study, was guided by a STAG in the field, using a robust method to research sites employing perinatal systems, drawing comparisons between them and identifying their preparedness to integrate active surveillance of pregnant persons into their existing platforms in LMICs.

A limitation of our study is that we included only six sites, when there are probably more potential sites for supporting active safety surveillance for pregnancy exposure. The sites were selected following a thoughtful methodological approach; however, the decision to include two sites per system was taken deliberately with the intention to pilot the assessment tool in different sites. Likewise, this study did not objectively evaluate the quality of the data reported by these sites nor the timeliness of the process which could be crucial when contributing to active safety surveillance.

## Conclusion

These findings could contribute to the successful implementation of active safety surveillance of vaccines during pregnancy by identifying and using active individual maternal and newborn data collection systems in LMICs.

## Supplementary Information

**Additional file 1. **Maternal, Newborn andChild Health variables under surveillance

## Data Availability

All data generated or analyzed during this study are included in this published article and its supplementary information files.

## Abbreviations

LMICS: Low- and middle-income countries
MNCH: Maternal neonatal and child health
GAIA: Global Alignment of Immunization Safety Assessment in pregnancy
WHO: World Health Organization
GNMNHR: Global Network’s Maternal Newborn Health Registry
INDEPTH: International Network for Demographic Evaluation of Populations and their Health
DHIS 2: District Health Information Software 2
ICD10: The tenth revision of the International Classification of Diseases
VPASS: Vaccine during pregnancy active safety surveillance
