# Supplementary material for: Data collection systems for active safety surveillance of vaccines during pregnancy in low- and middle-income countries: developing and piloting an assessment tool (VPASS)
Source: BMC Pregnancy Childbirth. 2023 Mar 13;23:172. doi: 10.1186/s12884-023-05417-8 (PMC10010225; doi:10.1186/s12884-023-05417-8)
Supplement: Supplementary file 1 — Additional file 1. Maternal, Newborn andChild Health variables under surveillance [file 12884_2023_5417_MOESM1_ESM.docx]

**Supplementary Material.**

Maternal, Newborn and Child Health variables under surveillance

|  |  |  |  |
| --- | --- | --- | --- |
| **Maternal variables** |  |  |  |
| **Variable** | **1: DHIS2** | **2: GNMNHR** | **3: INDEPTH** |
| **Maternal death** | Yes | Yes | Yes |
| **Preeclamsia/Eclampsia** | Yes | Yes | No |
| **Gestational hypertension** | Yes | Yes | Yes |
| **Foetal distress** | No | Yes | Yes |
| **Ectopic pregnancy** | No | Yes | No |
| **Postpartum hemorrhage** | Yes | Yes | Yes |
| **Spontaneous Abortion** | Yes | Yes | Yes |
| **Antenatal bleeding** | No | Yes | Yes |
| **Dysfunctional labor** | Yes | Yes | No |
| **Foetal growth retardation** | Yes | Yes | No |
| **Gestational diabetes** | Yes | Yes | No |
| **Post-partum endometritis** | No | Yes | No |
| **Chorioamnionitis** | No | Yes | No |
| **Premature preterm rupture of membranas** | No | Yes | No |
| **Preterm labor** | No | No | No |
| **Insufficient cervix** | No | No | No |

| **Neonatal variables** |  |  |  |
| --- | --- | --- | --- |
|  |  |  |  |
| **Variable** | **1: DHIS2** | **2: GNMNHR** | **3: INDEPTH** |
| **Neonatal death** | Yes | Yes | Yes |
| **Congenital anomalies** | Yes | Yes | Yes |
| **Neonatal infections** | Yes | Yes | Yes |
| **Preterm birth** | Yes | Yes | Yes |
| **Stillbirth** | Yes | Yes | Yes |
| **Low birth weight** | Yes | Yes | Yes |
| **Small for gestational age** | Yes | Yes | Yes |
| **Neonatal encephalopathy** | No | No | No |
| **Respiratory distress** | Yes | Yes | No |
| **Failure to thrive** | No | No | No |
| **Microencephaly** | No | No | No |
| **Neonatal seizures** | No | Yes | No |
| **Neurodevelopmental delay** | No | No | No |
